# The FrEEIA readiness assessment tool: an evidence-informed pro-equity readiness assessment tool adapted in Aotearoa New Zealand for the implementation of health interventions

**DOI:** 10.3389/frhs.2026.1733685

**Published:** 2026-03-19

**Authors:** Nina Veenstra, Papillon Gustafson, Michelle Lambert, Lisa Kremer, Holly O'Loughlin, Karen Bartholomew, Peter Carswell, Mihi Ratima, Adam Fusheini, Patricia Priest, Sue Crengle

**Affiliations:** 1 Ngāi Tahu Māori Health Research Unit, University of Otago, Dunedin, New Zealand; 2 School of Pharmacy, University of Otago, Dunedin, New Zealand; 3 Planning, Fundings and Outcomes, Health New Zealand, New Zealand; 4 Impact4Good Ltd, Auckland, New Zealand; 5 Taumata Associates Ltd, Hāwera, New Zealand; 6 Preventive and Social Medicine, University of Otago, Dunedin, New Zealand

**Keywords:** Aotearoa New Zealand, health equity, implementation science, Indigenous, Māori, readiness assessment

## Abstract

**Introduction:**

Equitable implementation is an important dimension of effective implementation. In Aotearoa New Zealand, many health interventions with the potential to lessen health inequities for Māori fail to do so because of implementation challenges. Equity readiness can ensure organisations are both willing and able to implement or scale up health interventions in a way that doesn't result in further health disparities.

**Methods:**

An equity readiness assessment tool, designed to be used by healthcare organisations in conjunction with an equity focussed process framwork (FrEEIA: Framework for Effective and Equitable Implementation in Aotearoa), was developed through a seven stage, mixed methods, iterative process. This tool frames equity readiness as a collective, multi-level construct. Initial stages of its development included interviews with interest holders to explore barriers and facilitators impacting the implementation of interventions to improve health equity, a reveiw of existing equity assessment and change readiness tools, and a researcher workshop to develop key domains. This was followed by the actual development/adaptation of a suitable tool with advice from interest holders who had utilised similar tools, testing and the development of additional resources that would aid its use.

**Results and discussion:**

The final version of the FrEEIA Readiness Assessment Tool is an adapted version of The Readiness Thinking Tool®, comprising 31 statements in three sections (individual readiness, intervention-specific readiness, and organisational readiness) which users rate individually and then discuss as a team, before formulating an action plan to improve equity readiness. Pilot testing highlighted the particular benefit of the tool in increasing awareness of the different dimensions of equity readiness, with the identification of strategies to address barriers to readiness more challenging due primarily to timing, team make up, and facilitation challenges. A range of supporting resources -a User Guide, Facilitator's Guide and Action Plan template- were developed to facilitate action plan development.The FrEEIA Readiness Assessment Tool is now available for use and adaptation through an interactive online interface, a format which was found to carry distinct advantages for tailoring feedback. The research team will continue to make refinements as this tool gets rolled out in a wider variety of service settings.

## 1 Introduction

Evidence-based healthcare interventions have great potential to contribute to improved health outcomes for all, if implemented effectively and with an intentional equity focus. While there is a significant time lag in the implementation of interventions, with the oft-cited figure being 17 years for research to translate to patient benefit (1), ineffective implementation also delays realisation of benefits. Equitable implementation is increasingly being recognised as an important dimension of effective implementation (2).

In Aotearoa New Zealand (NZ), many health interventions with the potential to lessen health inequities for Māori (the Indigenous peoples) fail to do so because of implementation challenges. For example, the consequences of inequitable implementation mean Māori have lower survival rates than non-Māori for almost all common cancers (3), due to inequitable access to and through the continuum of cancer care services (4). For cancers with active screening programmes, differential access to screening can alone account for much of the difference in survival (5). Decades of research have documented the range of practical (e.g., costs, transport, time), and other health systems barriers Māori face in accessing health services including the nature of interactions with staff and organisational structures i.e., structural racism (6). Equitable implementation, therefore, requires pro-equity planning and should consider the types and levels of resourcing and different approaches required for any intervention to achieve equitable outcomes (7).

In the same way that effective implementation is contingent on high levels of organisational readiness for change (8), so is equity readiness imperative to ensure equitable implementation. In this context, readiness implies being both willing and able to do what is being proposed (9). It therefore comprises multiple, dynamic components relating to, firstly, the motivation of individuals, secondly, the specific capacities required for the intervention in question, and lastly, the more general capacity of the organisation implementing the intervention (10). For the purposes of analysis, there are other aspects of readiness that should also be considered (11), including that it applies at different levels (individual, team, organisation, or even coalition) and can vary between these, as well as change over time in both positive and negative ways. Encouragingly though, readiness can be built if appropriate strategies are identified and acted on.

Currently there are a range of tools available to help organisations in the health sector assess their readiness for change (12–17). Furthermore, there are organisational self assessment tools focussed on equity (18–23). However, in Aotearoa NZ there was, at the time this research was initiated, no equity readiness tool that could be used by organisations in the health sector to assess their willingness and ability (i.e., readiness) to implement an intervention in an equity-enhancing way. Such a tool, that combines the concept of readiness with equity concerns, was therefore considered necessary to guide the equitable implementation and scale up of mainstream health interventions in the local context.

Work on the development of an equity readiness tool was undertaken through a collaboration between University researchers and the health service in Aotearoa NZ which aimed to develop implementation science tools with an equity focus that can be used in mainstream health services. They key output of the collaboration was an implementation process framework (24)—the Framework for Effective and Equitable Implementation in Aotearoa or FrEEIA, pronounced free-ah, which along with supporting tools is available online (www.impsciaotearoa.org.nz). In this framework, the foundation for action is enacting Te Tiriti o Waitangi (the legal agreement between Māori Rangatira/chiefs and the British Crown) principles, with whānau/extended family-centred implementation at its core.

This paper reports more specifically on research to develop an equity readiness assessment tool to assist with the ‘Implementation planning’ and ‘Designing the implementation pathway’ stages of the FrEEIA framework. The goal of this tool is to support individuals and organisations to reflect on factors influencing equitable implementation and to develop strategies to remove any barriers across a broad range of services and interventions, both planned and existing. Should these strategies be successful, services and interventions should be able to demonstrate more equitable outcomes. This tool therefore also complements the He Pikinga Waiora (HPW) Framework, an existing implementation framework developed in Aotearoa NZ for community-identified solutions and with a specific focus on co-design (25, 26).

For the purposes of the FrEEIA implementation science tools, equity is understood to be an ethical concept relating to health disparities that are unfair and unjust (7, 27). It is also consistent with human rights principles and the right to ‘the highest attainable standard of health’ (28). Practically this can be benchmarked by looking at the standard of health achieved by the most socially advantaged groups in society. In Aotearoa NZ, the right of Māori to this standard of health is protected under Te Tiriti o Waitangi (7), with equity one of the Treaty principles identified by the Waitangi Tribunal for the primary health care system (29). Nonetheless colonisation and systemic racism continues to result, through various processes, in unequal health outcomes for Māori (30, 31). The focus of the FrEEIA tools is therefore to ensure that implementation processes are effective and equitable, in order to achieve equal outcomes.

## 2 Materials and methods

The aim of this research was to develop an equity readiness assessment tool that could be used by healthcare organisations in conjunction with the framework to inform readiness for equitable implementation or scale-up of health interventions in Aotearoa NZ. In this paper the term ‘intervention’ is used to refer to a range of evidence-based improvements in the health sector comprising one or more of ‘the 7ps’: programs, practices, principles, procedures, products, pills and policies (32).

Consent and approvals: Ethical approval for this research was obtained from the Health and Disability Ethics Committee in New Zealand (ref 2023 EXP 15377). Research was conducted in accordance with ethical principles outlined in the Declaration of Helsinki and informed consent was obtained from all particpants prior to them completing surveys, being interviewed or participating in field testing.

Culturally safe approach (Māori oversight): Specific measures were taken in this research to best-ensure that the research process was not only culturally safe, but that the resulting tool would also address implementation readiness for Māori. The project was led by a senior Māori health researcher, while other research team members included Māori community or university-based researchers and the leader of a national Māori primary health care organisation. The research was guided by a project Kāhui—a grouping of Māori experts, some with expertise in data governance, and others representing the consumer perspective. It also had input from a Stakeholder Group, comprising 19 senior level health service managers from the public and non-governmental sectors. Both these groups were consulted at strategic points in the research and tool development process and their feedback included in the development and finalisation of the tools and associated documents.

This research was undertaken through an iterative mixed methods approach in seven stages including:

1. Interviews with key interest holders to explore barriers and facilitators impacting the implementation of interventions to improve health equity.
2. A review of existing equity assessment and change readiness tools
3. A researcher workshop to develop key domains for the readiness assessment tool and review of evidence in the use and design of self-assessment tools
4. Interviews with key interest holders to explore experiences in using equity tools
5. The development of the readiness assessment tool
6. Pilot testing of the readiness assessment tool
7. Final revisions of the readiness assessment tool and development of supporting resources

### 2.1 Interviews with key interest holders: barriers and facilitators

Semi-structured qualitative interviews were held with interest holders working across the continuum of designing and implementing interventions to improve equity. The goal of these interviews was to explore barriers and facilitators that could impact the equitable implementation of interventions, recognising that interventions intentionally designed with these in mind would be more likely to achieve equitable health outcomes. Sampling was purposive, with industry experts and researchers approached to participate on a voluntary basis. There were no specific inclusion or exclusion criteria, but a key focus was on significant representation of Māori and Pacific interest holders. Those interviewed included individuals working in service and management roles in then District Health Boards (now Health New Zealand), Māori health leads, general practitioners and managers/directors of non-governmental organisations.

Interviews were recorded, transcribed, and coded inductively, guided by Gioia et al.'s (33) approach. Four researchers were engaged in this process which culminated in a group workshop where third order domains were refined and reduced, with a definition developed for each.

### 2.2 Review of existing equity assessment and change readiness tools

Due to the importance of change readiness as a key part of successful implementation, a brief review of existing equity assessment tools and change readiness tools was undertaken. Google Scholar was used to identify in the first instance any systematic reviews of such tools, and secondarily, individual tools for review. Only papers in English were included and priority was given to tools used in a health context. Each tool was summarised in terms of its domains, items or questions, and the instructions or approaches to completing the tool. A list of all domains covered by the tools was compiled. Individual tools were then mapped against this aggregate list.

### 2.3 Researcher workshop to develop key domains for the readiness assessment tool and review of evidence in the use and design of self-assessment tools

Members of the research team met to develop the key domains for the equity readiness assessment tool. In attendence were researchers with expertise in health equity, Māori health, organisational psychology and implementation science, and the Aotearoa NZ health system. Discussions were based on the outputs from the previous two steps and all research team members present at the workshop reached a consensus on the domains that should be included in an equity readiness assessment tool for the Aotearoa NZ context.

In addition to deciding on domains, the team also considered best practice design principles that would make a readiness assessment tool user friendly and hence more likely to be used, while also increasing the likelihood of follow-on actions. This was done through a review identifying the most important design principles.

### 2.4 Interviews with key interest holders: experiences with equity tools

Interviews were held with interest holders working on health equity issues and with experience using equity tools. Sampling for these interviews was again purposive, with interest holders approached to be involved. All participation was voluntary and the interviews focussed on users’ experience with the language, structure and design of equity tools and the type of information that would be helpful in a user guide. Interviews were transcribed and coded deductively according these specific areas of interest.

### 2.5 The development of the readiness assessment tool

Domains and design principles were combined into an equity readiness assessment tool which could be piloted. The aim was to produce a tool capturing the domains and best practice design principles identified, while also ensuring that the tool and associated process for completion would be quick and easy enough to ensure completion.

### 2.6 Pilot testing of the readiness assessment tool

The FrEEIA readiness assessment tool was pilot tested in the field. The aim of this testing was to identify any futher modifications required to the tool itself and to inform the development of a user guide. Lung cancer screening teams were initially invited through their managers to test the tool, being ideally placed for participation because implementation planning for this programme is underway in Aotearoa NZ and the programme, if well implemented, has the potential to significantly contribute to improved health outcomes for Māori (34). The managers gave consent for teams to be contacted, with participation remaining voluntary for individuals. In addition, since planning for lung cancer screening was was still in the early stages, the invitation was also extended to other teams working on existing health programmes with an equity focus and known to the research team. Those consenting to parrtcipate in the pilot testing received an introductory session on the tool. They then went through the recommended process of completing it individually first, followed up by a facilitated team discussion of their responses led by an external facilitator.

Participants involved in pilot testing included clinical specialists, nurses and programme managers. As expected, teams exhibited different power dynamics and contrasting perspectives were voiced in some team discussions—the external facilitator ensured that all voices were heard while mainting a safe environment. Participants were invited to complete a survey to determine how they found the equity readiness assessment process, as well as given the opportunity for a recorded, semi-structured interview to discuss their responses in greater detail. Feedback, mostly of a qualitative nature, focussed on the clarity and length of the tool, its strengths, weaknesses and limitations, and any improvments that could be made. Survey data were collected and managed using REDCap electronic data capture tools hosted at University of Otago (35, 36). Facilitated discussion sessions were observed, recorded and transcribed. Two researchers coded the interviews and observations deductively and independently according to a codebook, using NVivo Qualitative Research Software (37). This coding extracted feedback on various elements of the tool and the readiness assessment process. Coding discrepancies were discussed and resolved by members of the research team through consensus.

### 2.7 Final revisions of the readiness assessment tool and development of supporting resources

Pilot testing informed a further round of revisions of the tool, as well as drafting of the associated documents—a User Guide, a Faciltiator's Guide and an Action Plan template.

## 3 Results

**Figure 1:**
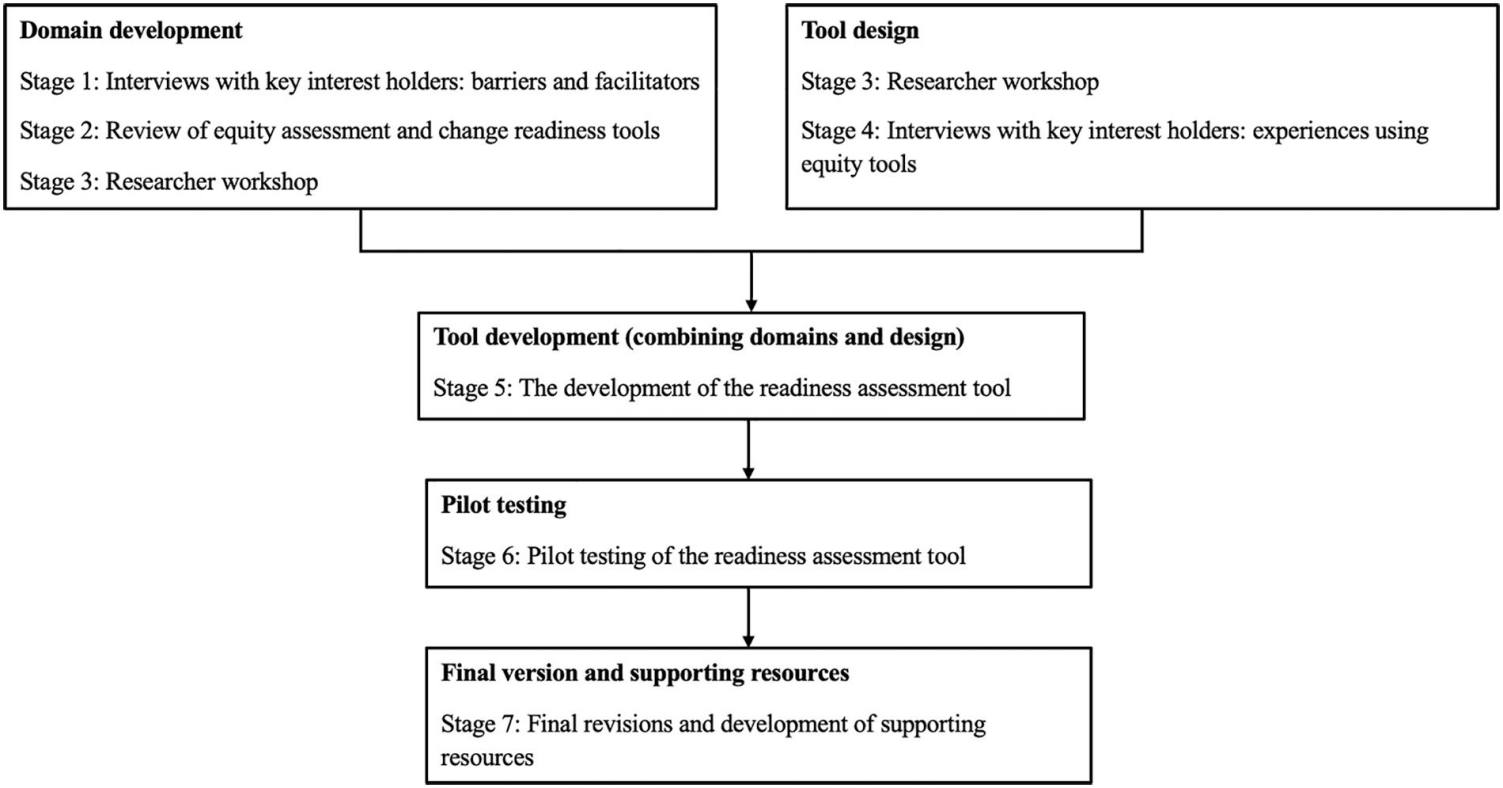
Stages of the research feeding into development of FrEEIA readiness assessment tool.

Figure 1 illustrates how various stages in the reseach contributed to the development of the FrEEIA readiness assessemnt tool.

### 3.1 Stages 1, 2 and 3: domain development

Tool domains were developed in stages one, two and three. In stage one, 25 interviews lasting 45–60 minutes were undertaken. Analysis found 16 themes that could be considered as potential domains for the FrEEIA readiness assessment tool. Stage two reviewed 12 tools, with 10 common domains identified. An additional text file gives more details about these tools [see Additional File 1]. Stage three resulted in a list of 14 potential domains for inclusion in the FrEEIA readiness assessment tool. The example of the domain ‘Partnership’ can further illustrate how each was developed. In this case partnership was identified through interest holder interviews as a pro-equity determinant of implementation success. While it was then not an explicit domain in the equity assessment or change readiness tools identified in stage two, its significance in the stage one data, as well as its positioning as a Treaty principle recommended for use by the primary health system (29), warranted its inclusion as a new domain in the Organisational Readiness section of the FrEEIA tool.

Table 1 shows the potential domains identified in stages one and two, and the outcome of stage three.

**Table 1:** Potential domains for inclusion in the FrEEIA readiness assessment tool.

Potential domains identified through qualitative interviews	Potential domains identified from existing equity assessment and change readiness tools	Potential domains identified through researcher discussion
• Policy • Funding • Partnership • Workforce and training • Values/attitudes/beliefs • Agreement on appropriateness • Championship • Compatibility • Leadership • Collaborative design • Learning and improvement • Equity outcomes • Team dynamics • Organisational culture • Communication • Adequate resources	• Policies/processes towards equity • External collaboration • Workforce and leadership • Relevance of the need for the intervention • Organisational alignment and compatibility • Organisational climate/culture • Quality improvement and quality standards • Language and communication access • Access • Resources/infrastructure	• Policy/funding context • Partnership • Workforce/training • Leadership support • Relevant evidence • Values/attitudes/beliefs • Pro-equity • Racism/de-colonisation • Organisational culture • Alignment/fit with organisational context • Quality/standards • Communication • Access • Adequate resources

### 3.2 Stages 3 and 4: tool design

The actual design of the tool according to best practice principles was accomplished during stages three and four of the research.

The researcher workop in stage three, in addition to looking at domains, also involved a broad review of a range of documents (38–42). Stage four, comprising eight interviews with interest holders working on health equity issues and with experience using equity tools, identified further best practice principles for designing and using a self-assessment tool.

Such principles included the need for:

- a tool that is adaptable
- a tool that is relatively short and quick to complete
- a preamble outlining the evidence base for the tool
- domains/components that are meaningful and relevant to those who would be using the tool
- simple and strength-based language
- a measurement scale that is sensitive enough to allow follow-on conversation (ie more than just yes/no)
- management of power imbalances between team members. In particular, it was deemed important that the tool be completed individually first. Individual completion, as well subsequent team discussion, would also ideally be supported through external facilitation
- inclusion of action planning and additional resources to support actions.

### 3.3 Stage 5: tool development (combining domains and design principles)

The result of stage five, the actual development of an appropriate tool, was that the FrEEIA readiness assessment tool was ultimately based on the format of ‘The Readiness Thinking Tool®’ (14), as it fulfilled many of the best practice principles identifed. The decision to adapt an existing tool came only after an initial attempt to develop an entirely new tool on from the bottom up. In the first instance, the research team used their set of first 14 domains (see Table 1) to develop a tool with 50 statements. In this first version of the tool, each statement was ranked against two 5-point scales focussing on importance and feasibility. While comprehensive, feedback on this tool from both researchers and industry interest holders was that this level of detail may ultimately result in non-completion.

Domains in The Readiness Thinking Tool® (Wandersman Center) tool were closely aligned to those identified in this research and completion was deemed less onerous, so adaptation (with permission from the authors) evolved as a better option. The original tool is available under a Creative Commons license and has three sections (motivation, innovation-specific capacity, and general capacity) and 19 domains/statements that are measured on a simple 3-point scale (if the area is a strength, challenge, or unsure). While more recent research has considered how to make The Readiness Thinking Tool® more user friendly (43), in our adaptation section names were changed to read: individual readiness, intervention-specific readiness, and organisational readiness. An additional twelve statements were then added exploring issues identified as relevant to equitable implementation during stages one to three of our research (see Table 2). Like the original tool, individuals can decide whether and to what extent they agree with the different statements. Individual responses are then used as the basis for dicussions between members of the implementation team. Unlike the original tool, the FrEEIA equity readiness assessment includes an additional action planning step.

**Table 2:** Comparison of statements/domains in the adaptation of the readiness thinking tool® (wandersman center).

The Readiness Thinking Tool® (Wandersman Center) domains/statements	FrEEIA domains/statements
MOTIVATION	INDIVIDUAL READINESS
Relative advantage Compatability Simplicity Ability to pilot Observability Priority	Relative advantage Alignment Evidence* Flexibility Outcomes* Priority
INNOVATION SPECIFIC CAPACITY	INTERVENTION-SPECIFIC READINESS
Innovation specific skills and knowledge Champion Supportive climate Inter-organisational relationships Intra-organisational relationships	Skills and knowledge Championship Supportive work environment Service user engagement User centered design Internal organisational relationships External organisational relationships Adaptation
GENERAL CAPACITY	ORGANISATIONAL READINESS
Culture Climate Innovativeness Resource utilisation Leadership Internal operations Staff capacities Process capacities	Māori (Indigenous) leadership* Local leadership Equity leadership* Organisational culture Training* Knowledge* Innovativeness Partnership* Resource use Staff capacities Data* Improvements Process capacities Communication*

*New question area.

Initial feedback from the research team on the adapted FrEEIA readiness assessment tool centred primarily around the need to define an appropriate measurement scale for responses to each statement, and modifications to make the language accessible and user-friendly. During the iterative process of revision and redrafting, the tool also became progressively more focused on individual and organisational factors affecting implementation, rather than broader contextual factors, which interest holders felt teams would have less power to influence through their action plans.

### 3.4 Stage 6: pilot testing

Stage six, pilot testing of the FrEEIA readiness asessment tool, was done with four implementation teams comprising a total of 24 people. It revealed that overall, participants were happy with the time commitment associated with completing the tool and the majority felt it gave them a better understanding of equity readiness. They suggested it ‘made them think’, especially about elements that they hadn't previously considered, it was a good teambuilding exercise and a useful tool to ensure that ‘everybody is on the same page’. Many potential positive impacts were identified, including that the process could give a team confidence about their program, by ensuring issues identified could be addressed early in the implementation process. The results could be used to motivate for additional support or resources, or monitor progress/improvements if done repeatedly, especially when this progress is not yet reflected in outcome measures.

In terms of negative impacts, participants suggested it could be confronting for a team to find out there is disagreement within the team and disheartening if there were many red flags and teams came to realise that they were not as prepared as they should be. The tool also had the potential to create disunity in a team if there were disagreements about what needs to change, or nothing got done because of the process, resulting in unmet expectations. Attention was given to ways of mitigating these risks, with preparation of the team and skilled facilitation viewed as key, to ensure everyone participating in the process is prepared, disagreement can be viewed in a positive light, and expectations are met.

In pilot testing, teams found using the outcome of discussions to formulate an action plan the most challenging part of the readiness assessment process. Close to half of those completing the survey felt that at the end of the process, their team hadn't successfully planned changes based on the outcome of the readiness assessment. Further investigation as to the reasons teams found this step challenging pointed to issues related to: timing and team make up, understandings around the implementation pathway and where a teams’ sphere of influence lies, as well as the style of facilitation used during the team discussion. Teams made up of only clinicians often found themselves ’stuck’ on organisational readiness domains that they had little control over, highlighting the need for health service teams going through the process to be diverse in their make up (including clinicians, administrators and managers). Teams implementing interventions in earlier stages of planning found it hard to visualise where they sat on the implementation pathway, requiring some additional groundwork that facilitators weren't prepared for. Furthermore, it became clear that an example action plan would have helped all teams to visualise where they were going and what the output would look like.

### 3.5 Stage 7: final revisions and development of supporting resources

Pilot testing findings informed stage seven of the research, which involved final revisions of the FrEEIA readiness assessment tool itself and the compilation of supporting resources, including a Facilitator's Guide and an Action Plan Template. Initially the research team had identified the need for a User Guide, but it became apparent during pilot testing that a website could house much of the information that would be in the User Guide and that teams required more targeted support in the process of developing an action plan, including the facilitation required to achieve this. Only minor further changes were made to the tool itself, incorporating suggestions to make wording clearer. The key focus of stage seven was on the development of supporting resources and a website to house the interactive tool. (www.impsciaotearoa.org.nz). The final tool, comprising 31 statements and associated discussion questions is now available for use and adaptation under a Creative Commons license. It can be viewed in hard copy format on the website along with the Facilitator's Guide, which takes interested parties through the recommended steps for using the tool interactively and understanding what the whole readiness assessment process involves. A copy of FrEEIA readiness assessment tool statements is included in Table 3.

**Table 3:** FrEEIA readiness assessment tool statements.

Domain name	Domain statement
Section 1: Individual readiness	
Alignment:	This intervention fits with how I usually do things around here
Benefit:	This intervention is as good as (or better) than what I am currently doing
Evidence:	The evidence for how this intervention will improve equity is clear
Flexibility:	This intervention can be adapted to my local context
Outcomes:	I can see how this intervention will lead to improved equity outcomes
Priority:	Getting this intervention working is a priority amongst other things I need to do
Section 2: Intervention-specific readiness	
Skills and knowledge:	We have sufficient skills and knowledge to implement the intervention
Leadership champion:	We have someone in a leadership role, or a well-connected person, who supports and will advocate for this intervention
Supportive work environment:	We have the necessary supports, processes, and resources to enable this intervention
Service user/community engagement:	There are mechanisms for service users, communities and those most impacted (people at highest risk and/or with lived experience) to be meaningfully involved in implementation
User-centred design:	Services are designed to meet the needs and preferences identified by those impacted by inequities
Internal organisational relationships:	The relationships within this organisation support the intervention
External organisational relationships:	The external relationships necessary for this intervention to work exist and are supportive of the intervention
Pathway design:	The implementation pathway: a) Considers the context where the intervention will be delivered, including any adaptations to address equity barriers (e.g., access), and b) Establishes the relevant monitoring and evaluation metrics
Section 3: Organisational readiness	
Staff capacities:	We have enough of the right people to get things done
Staff characteristics:	Our workforce reflects the population served, in particular the communities that experience the greatest inequities
Knowledge:	Staff are knowledgeable about inequities and their causes, and have a shared language and understanding
Māori leadership:	We have Māori in leadership roles in our organisation
Local leadership:	We have people from the population served who have leadership roles in our organisation
Equity leadership:	Leaders in our organisation actively promote equity and ensure accountability for equity action
Culture:	Equity is a part of the norms and values in how we do things here
Innovativeness:	There is an openness to do things differently to address inequities
Communication:	Language and communication needs and health literacy demands are explicitly considered in: a) Collateral resources (e.g., brochures, letters, videos, graphics, maps), and b) Interactions between service users and staff (in any role)
Partnership:	There are clear and visible partnerships between the service/organisation and communities that experience inequities
Training:	There is training or specific programmes available to ensure foundational knowledge, awareness and tools to act on inequities, e.g., Te Tiriti, decolonisation, anti-racism, cultural safety
Data:	There are systems to measure and monitor data (access, quality, outcomes) by relevant equity parameters, e.g., ethnicity
Improvements:	We use local data to identify and prioritise areas of focus to address inequities
Process capacities:	We have the ability to robustly plan, implement, and evaluate changes in areas that effect equity
Resource use:	There is the ability to acquire and allocate resources, including time, money, effort, and technology for equity-focused work

The Facilitator's Guide was designed to be used by the person (recommended to be external to the team) who is designated to facilitate the equity readiness assessment process for the implementation team. It aims to ensure that facilitation of the group discussion is done in a way that is culturally safe and effective, while guiding discussions towards potential actions. For example, the guide looks at how to use the results of online tool completion to focus discussion and how to determine where the team's sphere of influence lies. The Action Plan template includes a set of tables that can be completed following discussions to clearly document how barriers will be addressed by the team, including timing and who might be responsible.

## 4 Discussion

This research set out to develop an equity readiness assessment tool that would be suitable for use in a wide variety of mainstream (as opposed to kaupapa Māori; by, with, and for Māori) health service contexts at all levels in Aotearoa NZ. The resulting tool is designed to inform readiness for equitable implementation or scale-up of health interventions to ensure equitable access for Māori and potentially other groups. The development of the tool was challenging because of the complex nature of health care organisations and the multi-level, multi-dimensional nature of readiness (44). Designing a tool that would work equally well in a small rural general practice or a large hospital department required careful consideration of both the domains to be included, the specific wording of statements, as well as the process to follow. The resulting tool, the FrEEIA readiness assessment tool, was pilot tested by four teams in the planning stages of implementing an intervention in a process which is expected to be ongoing as the tool gets rolled out in a wider variety of health service settings. The research, in addition to successfully developing a tool, further exposed equity readiness as relational, with the process of assessment educative and sometimes confronting.

The FrEEIA readiness assessment tool, like the Wandersman Centre tool (14) on which it is based, considers equity readiness as a function of being both willing (the psychological factors associated with readiness) and able (the structural factors associated readiness) (9). Historically, tools assessing change readiness have often been limited by their narrow focus on either psychological or structural factors (44), with a tendency towards assessing capacity for implementation in health (45). However health workers’ personal perceptions of the value of an intervention are independently associated with readiness at an individual and collective level (46). The FrEEEIA readiness assessment tool therefore advances many existing readiness assessment or equity tools through its focus on not only content, but also function, by taking health teams through a process that helps with collective sense-making and alignment. Specific components or domains were included in the tool based on qualitative research and tool review, lending weight to its evidence base.

Another strength of the FrEEIA readiness assessment tool is that it is available for use in different formats. During field testing, individuals entered their responses to readiness statements into REDCap (35, 36) and researchers manually prepared feedback for the team in graphical format, to act as a basis for facilitated discussion. Participants clearly indicated their preference for this type of tailored feedback, suggesting that the tool would be best presented using an interactive interface. The paucity of interactive readiness assessment tools for global health interventions has been previously noted and this limitation was evident too amongst the tools reviewed during this research (45). Hence this led to the development of the FrEEIA web-site (www.impsciaotearoa.org.nz), where the FrEEIA readiness assessment tool can be completed online and reporting at both an individual and team level is automated based on user input.

The identification or development of strategies for addressing barriers to implementation is known to be challenging both practically and conceptually (47, 48). In pilot testing of the FrEEIA readiness assessment tool, and in particular during the facilitated discussion component of the process, teams struggled most with moving from the identification of barriers to equitable implementation, to the point of pinpointing strategies to address these barriers. These struggles are reflective of readiness assessment as a sense-making and alignment exercise, where moving towards constructive action could conceivably require additional organisational supports. Nevertheless, the research team responded to this finding with a greater focus on action planning in the final tool version and supporting resources developed through the pilot testing. These will hopefully facilitate the action planning process, and future learning from the rollout of the tool will also help inform ways to make this step more straightforward to implementation teams.

It was clear during testing that the FrEEIA readiness assessment tool had much to offer in terms of getting teams thinking about equity and the many different factors that could impact equitable implementation. Overall, it helped to ensure that members of an implementation team were more united in their understanding of equity and in their commitment towards addressing inequities in the delivery of their service. In this sense, the importance of ‘enlightenment use’ (45, 49), the educative function of undertaking the process, should not be underestimated. Having implementation teams going through the equity readiness assessment process, even if change is not immediately apparent or teams find it hard to develop a plan, still carries substantial benefits. Furthermore, with repeated use of the FrEEIA readiness assessment tool, the process should become more familiar to the teams using it, making it easier for teams to develop and refine action plans. How often to repeat the readiness assessment process would be a decision for individual teams, however certain instances might call more strongly for this, such as when scaling up an intervention.

There is always a risk that in going through the FrEEIA readiness assessment process, racist comments might be made, particularly in teams that are not equity focused or are made up of people with limited cultural competence or safety. Although not encountered during our pilot testing, this can potentially put other members of the team in a culturally unsafe space. There is also the issue of white fragility and the range of emotional and behavioural reactions that this can generate (50). Such concerns have led the research team to strongly recommend external facilitation for the group discussion, by someone experienced in dealing with the kinds of comments or reactions that can arise during such a process, while also acknowledging this may be challenging in resource-constrained settings. The key requirements of an external facilitator would be that they are skilled in managing group dynamics, focussing discussions and maintaining a safe environment, and as such they could be recruited from many places. They would not need to have any prior knowledge of implementation science.

While a significant amount of work went into ensuring a sound evidence base for the FrEEIA readiness assessment tool, field testing to date has been limited. Experience suggests that it is difficult to test such a tool in a research environment, primarily because timing is critical to ensure relevance, and this can be difficult to achieve. With some of the teams participating in the pilot testing, it was clear that not all members were yet familiar with the implementation pathway and what their role in the delivery of the new intervention would look like. It is also challenging to get testing happening within all the diverse health settings for which the tool is designed. Hence the pilot testing done thus far should be viewed quite simply as the first step in an ongoing process of collating and responding to feedback from users via the web-based interface, which will likely result in further refinements and iterations of the tool and associated resources.

In addition to feedback via the web-based interface, the research team is also involved in more structured ongoing testing of the FrEEIA assessment tool, the current goal being to assess its use in a more diverse range of health service settings, including primary care and allied health services. There may also be other parties interested in its transferability to other groups or settings. While the tool was developed for the Aotearoa NZ context and with the explicit goal of addressing Māori health equity, all tool domains except ‘Māori leadership’ could be relevant for addressing inequitable implementation of health interventions for other minority groups. Tool statements for some other domains would need different wording though. For example, under ‘Training’ there are some recommendations that relate to Māori health equity specifically; Te Tiriti, decolonisation, anti-racism, and cultural safety. Hence while we don't specifically advocate for the tools’ use for other groups or in other contexts internationally, we acknowledge that with further research and adaptation it could find utility.

## 5 Conclusions

In Aotearoa NZ, evidence-based health interventions have the potential to contribute towards equitable health outcomes for Māori, but can fail to do so because of implementation challenges. Pro-equity implementation science tools that are responsive to the local context aim to improve intervention effectiveness and equity across a range of potential equity dimensions. The FrEEIA readiness assessment tool described in this paper was developed through a rigorous mixed-method seven-step process, including pilot testing with teams in four different settings. This testing affirmed the benefit of explicitly assessing equity readiness as a team and informed the further refinement of the tool and the development of accompanying resources. The FrEEIA readiness assessment tool is now available online for use in mainstream health services. It is also available under a Creative Commons license for adaptation, and the research team intend to continue making refinements as it gets rolled out in a wider variety of service settings.

## Data Availability

The datasets presented in this article are not readily available because this would not be in accordance with ethical consent provided by participants. Requests to access the datasets should be directed to the corresponding author.
