# Supplementary material for: The FrEEIA readiness assessment tool: an evidence-informed pro-equity readiness assessment tool adapted in Aotearoa New Zealand for the implementation of health interventions
Source: Front Health Serv. 2026 Mar 19;6:1733685. doi: 10.3389/frhs.2026.1733685 (PMC13044145; doi:10.3389/frhs.2026.1733685)
Supplement: Supplementary file 1 [file supplementaryfile1.docx]

**Additional file 1: Tools reviewed for this research**

- Implementation Guide for Programmatic Lung Cancer Screening (12)
- WHO Readiness Assessment Tool (13)
- R = MC ^2^ Readiness Thinking Tool ® (14)
- Comprehensive assessment of organizational functioning and readiness for change (ORC) (15)
- Organizational Readiness to Change Assessment (ORCA) tool (16)
- Organizational Change Readiness Assessement (17)
- Health Equity Assessment Tool (HEAT) (18)
- Culturally Effective Organizations (CEOrgs) Framework Organizational Assessment (19)
- Bay Area Regional Health Inequities Initiative (BARHII) Organizational Self-Assessment Tool (20)
- Race matters: organizational self-assessment (21)
- ABLe change Equity Organizational Self Assessment (22)
- Tool for Organizational Self-Assessment Related to Racial Equity (23).

1. Canadian Partnership Against Cancer. Implementation Guide for Programmatic Lung Cancer Screening 2020 [Available from: <https://www.partnershipagainstcancer.ca/topics/lung-screening-resources/>.

2. WHO. Readiness Assessment Tool 2018 [Available from: <https://cdn.who.int/media/docs/default-source/reproductive-health/contraception-family-planning/readiness-assessment-hexagon-tool.pdf?sfvrsn=e0270b5b_3>.

3. Wandersman Center. Readiness Thinking Tool ® [Available from: <https://www.wandersmancenter.org/uploads/1/2/8/5/128593635/531c7f_dbd8360b53b146b6a4fbffcb321cacfd.pdf>.

4. Lehman WEK, Greener JM, Simpson DD. Assessing organizational readiness for change. Journal of Substance Abuse Treatment. 2002;22(4):197–209.

5. Helfrich CD, Li Y-F, Sharp ND, Sales AE. Organizational readiness to change assessment (ORCA): Development of an instrument based on the Promoting Action on Research in Health Services (PARIHS) framework. Implementation Science. 2009;4(1):38.

6. Canada Health. Organizational Change Readiness Assessment 2012 [Available from: <https://www.infoway-inforoute.ca/en/component/edocman/governance-and-leadership/resources-and-tools/670-organizational-change-readiness-assessment?Itemid=103>.

7. Signal L, Martin J, Cram F, Robson B. The Health Equity Assessment Tool: A user’s guide. Wellington: Ministry of Health/Manatū Hauora; 2008.

8. New Hampshire Equity Collective. Culturally effective organisations framework – organisational assessment [Available from: <https://equitynh.org/culturally-effective-organizations/#:~:text=The%20Culturally%20Effective%20Organizations%20Framework,%2C%20and%20non%2Dprofit%20organizations>.

9. Bay Area Regional Health Inequities Initiative. Organizational Self-Assessment Tool [Available from: <https://healthequityguide.org/resources/barhii-organizational-self-assessment-tool/>.

10. The Annie E. Casey Foundation. Race matters: organizational self-assessment. Baltimore: The Annie E. Casey Foundation; 2006.

11. Foster-Fishman P, Watson E. ABLe Equity Organizational Self-Assessment: Michigan State University; 2017 [Available from: <https://systemexchange.org/application/files/2315/4327/2119/ABLe_EquityOrganizationalSelf-Assessment_F.pdf>.

12. Coalition of Communities of Color. Tool for Organizational Self-Assessment Related to Racial Equity. 2014.
